# Response mechanism of major secondary metabolites of *Polygonatum kingianum* to selenium nanoparticles

**DOI:** 10.3389/fpls.2024.1480079

**Published:** 2024-12-18

**Authors:** Xiaolin Wan, Jiehua Wang, Jiaxin Zhang, Hongshi Cui, Lingjun Cui, Qiang Xiao

**Affiliations:** ^1^ Hubei Key Laboratory of Selenium Resource Research and Biological Application, Hubei Minzu University, Enshi, China; ^2^ Hubei Key Laboratory of Biological Resources Protection and Utilization, Hubei Minzu University, Enshi, China

**Keywords:** selenium nanoparticles, *Polygonatum kingianum* coll. et Hemsl, secondary metabolism, transcriptome, metabolome

## Abstract

Selenium nanoparticles (SeNPs) can be absorbed by plants, thereby affecting plant physiological activity, regulating gene expression, and altering metabolite content. However, the molecular mechanisms by which exogenous selenium affects *Polygonatum kingianum* coll.et Hemsl plant secondary metabolites remain unclear. In this study, we exposed *P. kingianum* plants to SeNPs at 0, 10, 25, and 50 mg/L concentrations. Joint physiological, metabolomic, and transcriptomic analyses were performed to reveal the response mechanisms of major secondary metabolites of *P. kingianum* to SeNPs. Our data shows that under the treatment of 25 mg/L, the photosynthetic electron transfer rate of plants significantly increases and the carbon-nitrogen ratio significantly decreases. In parallel, the main active components, polysaccharides and saponins, showed a significant increase in content, while flavonoid content decreased. SeNPs affect polysaccharide accumulation mainly through up-regulation of SPS, UGPase, AGPase, UTP, and SUS genes in starch and sucrose metabolic pathways. The accumulation of saponins was affected by upregulating genes in the sesquiterpenoid and triterpenoid biosynthesis pathways, including PAD, ADH, PK, and GS. The accumulation of flavonoids was mainly regulated by metabolic pathways such as flavonoid biosynthesis, isoflavonoid biosynthesis, and the biosynthesis of phenylpropanoids. In summary, this study reveals the key metabolic pathways affected by SeNPs in the main secondary metabolic products of *P. kingianum*.

## 1 Introduction

Selenium, a non-metallic essential micronutrient, is pivotal in safeguarding human and animal health by enhancing enzyme activities such as selenase and peroxidase (Angamuthu et al., 2019; Mao et al., 2016). Recent studies have shown that selenium is vital in regulating muscle function, male reproduction, and cardiovascular, endocrine, neurological, and immune system functions (Hartikainen, 2005; Razaghi et al., 2021; Hadrup and Ravn-Haren, 2021). However, selenium content is unevenly distributed on Earth, with about 72% of the soil in China being selenium-deficient (Wang et al., 2021) and about 1 billion people worldwide being selenium-deficient (Nothstein et al., 2017). In recent years, selenium nanoparticles (SeNPs) have been introduced as stabilized selenium nano-forms for stress regulators and crop fertilizers (Djanaguiraman et al., 2018). Compared with conventional organic and inorganic selenium, SeNPs have the advantages of lower toxicity, high glutathione peroxidase activity, high thioredoxin reductase activity, and high catalytic efficiency (Wang et al., 2007). Studies have shown that applying SeNPs can improve crops' nutritional value (Mittler, 2017).

*Polygonatum kingianum* coll.et Hemsl is a perennial medicinal plant of Liliaceae, and its dried rhizomes are included in the 2020 edition of the Chinese Pharmacopoeia. *P. kingianum* rhizomes mainly contain polysaccharides, flavonoids, saponins, alkaloids, and other secondary metabolites, which have antidiabetic, antitumor, immune-enhancing, and kidney-protecting effects, making it one of the most commonly used herbal medicines (Lu et al., 2016; Khan et al., 2013). Previous studies have found that specific selenium concentrations benefit plant growth (Puccinelli et al., 2017). Moderate amounts of exogenous selenium can improve photosynthesis and mineral uptake in plants such as stevia (Borbely et al., 2021), rice (Zhao et al., 2023) and tomato (Hajlaoui et al., 2023). Selenium can also affect the expression of plant senescence-related genes and delay plant senescence through NO signaling (Hajiboland et al., 2019). Selenium also improves plant carbon and nitrogen metabolism, which helps plants cope with external stresses and improves plant growth (Bhadwal and Sharma, 2022). In addition, moderate concentrations of selenium can increase the content of secondary metabolites in plants. For example, moderate amounts of selenium can increase the content of total phenols and flavonoid compounds in lemon verbena (Ghanbari et al., 2023), as well as the content of phenols, flavonoids, and terpenes in the leaves of Aloe vera (Zou et al., 2020). However, high selenium concentrations can cause plant damage, even leading to mortality in severe cases (Hawrylak-Nowak et al., 2015). The above studies provide a basis for understanding plant responses to different selenium concentrations. However, the molecular mechanisms of how selenium affects secondary metabolites in the rhizomes of the Chinese medicinal herb *P. kingianum* need to be better understood. In order to promote the healthy development of the selenium-enriched *P. kingianum* industry, it is crucial to study in depth the regulatory mechanisms of selenium on the accumulation of secondary metabolites in *P. kingianum.*

## 2 Materials and methods

Three-year-old *P. kingianum* plants were used as experimental materials. Plants with stable growth, similar morphological structure, and size were selected from the *P. kingianum* nursery of the Key Laboratory of Conservation and Utilization of Biological Resources of Hubei Province, China. The plants were potted in plastic containers filled with 15 liters of vermiculite and peat mixture (8:1 ratio), one plant per pot, and allowed to grow for 60 days. During this period, water once every 3 days with 500ml of tap water each time.

Four selenium nanoparticles (SeNPs) levels were set at 0, 10, 25, and 50 mg/L, designated as CK, Se10, Se25, and Se50, respectively. Each treatment had five biological replicates. SeNPs were produced by the Hubei Academy of Agricultural Sciences (≥80% selenium, particle size 40 to 270 nm). By root application, 250 ml of SeNPs solution (0.354 ml/cm^2^) was uniformly poured into the soil centered on *P. kingianum* plants. After 40 days of treatment, samples were taken to determine the parameters.

### 2.1 Determination of selenium content

The total selenium content in the rhizomes and leaves of *P. kingianum* under different treatments was determined by hydride atomic fluorescence spectrometry (GB 5009.93-2017).

### 2.2 Chlorophyll fluorescence parameters

Chlorophyll fluorescence parameters of *P. kingianum* leaves were measured using a Mini PAM portable chlorophyll fluorometer produced by the German company Walze. Leaves were selected from the middle of the main stem of the plant and dark-adapted for 20 min before each measurement. Initially, measuring light (< 0.1 μmol m^-2^ s^-1^) was shone, followed by saturating pulse light (3000 μmol m^-2^ s^-1^) to measure the initial fluorescence (Fo) and maximum photochemical quantum yield (Fv/Fm). After the initial fluorescence (Fo) and maximum photochemical quantum yield (Fv/Fm) were determined, the endogenous photochemical light (33 μmol m^-2^ s^-1^) was turned on, and the far-infrared light was irradiated at steady state to determine the apparent photosynthetic electron transfer rate (ETR), photochemical quenching coefficient (qp), and non-photochemical quenching coefficient (NPQ). The above parameters were read directly from the instrument. Finally, the potential activity of PSII (Fv/Fo) and the actual photochemical efficiency of PSII (*Φ*PSII) were calculated from the measured values of each. Six leaves were measured for each treatment.

### 2.3 Carbon and nitrogen metabolism

The rhizomes and leaves of *P. kingianum* were dried and ground to pass through a 100-mesh sieve. The carbon and nitrogen contents were determined using an elemental analyzer, the Vario MACRO cube (ELEMENTAR, Germany). Three replicates were set up for each treatment.

### 2.4 Determination of main secondary metabolites content and metabolomics analysis

Based on the results of previous studies, the major secondary metabolites in *P. kingianum* were identified as polysaccharides, flavonoids, and saponins. The saponin content was determined by following the basic scheme of Zhang et al. (Zhang et al., 2019). with the following modifications: 0.5 g of *P. kingianum* rhizome or leaf powder dried to a constant weight at 60 °C was weighed with precision. The material was extracted with 7.5 mL of petroleum ether using ultrasound for 30 min; after removing the solvent from the residue, 20 mL of 80% ethanol was added, and ultrasonic extraction was performed for another 30 min; 15 mL of n-butanol was used to evaporate the solvent, and the filtrate was filtered. evaporate the solvent; the residue was suspended in 2.5 mL of 0.5 mol L^-1^ HCl, followed by ultrasonic extraction for 30 min; 15 mL of n-butanol was added; the butanol was evaporated; and the residue was made up to 7.5 mL with methanol to obtain the test solution. Thirty microliters of each test solution were mixed with 0.2 mL of 5% vanillin-ice acetic acid solution and 0.8 mL of perchloric acid, incubated in a 60 °C water bath for 25 min, then cooled in an ice bath for 2 min, followed by the addition of 5 mL of ice acetic acid, mixed well, and allowed to stand for 5 min; the absorbance of the reaction solution was measured at 550 nm, repeated three times. The total saponin content was calculated based on the standard curve.

The polysaccharide content was determined following the basic protocol of Dubois et al. (DuBois et al., 1956). with the following modifications: *P. kingianum* powder (0.05 g) dried to constant weight at 60 °C was weighed accurately, 80% ethanol was added, heated and refluxed for 1 h, and the filtrate was evaporated; the residue was extracted with 30 mL of distilled water by reflux heating for an hour. The resulting filtrate was made up to 50 mL with distilled water to prepare the test solution. One hundred microliters of the test solution were mixed with 1 mL of 5% phenol, followed by the addition of 5 mL of concentrated sulfuric acid, mixed well, and incubated in a boiling water bath for 30 min. After cooling to room temperature, absorbance at 490 nm was measured in a UV-visible spectrophotometer (TU-1901) and repeated 3 times.

The determination of flavonoid content followed the basic protocol of Rofiqah et al. (Rofiqah et al., 2022). with the following modifications: 0.4 g of rhizome powder and 0.2 g of leaf powder of *P. kingianum*, both dried at 60 °C to a constant weight, were accurately weighed. The total flavonoids of *P. kingianum* were extracted using a lipid meter (Hainergy Technology, SOX406). The weighed powder was placed in a filter paper cartridge and put into a filter paper holder, which was held in place by a magnet. 10 mL of 70% ethanol was added to the extraction cup, so that the filter paper was immersed in ethanol, extracted at 98 °C for 30 min, followed by Soxhlet extraction for 30 min, and the filtrate was retained. The extraction process was repeated, and the filtrates were combined into 20 mL to obtain test solutions for rhizomes and leaves. Two milliliters of the test solution were mixed with 0.3 mL of 5% sodium nitrite (mixed and left to stand for 6 min), 0.3 mL of 10% aluminum nitrate (mixed and left to stand for 6 min), and 2 mL of 4% sodium hydroxide (mixed and left to stand for 10 min); the absorbance of the reaction solution was measured at 510 nm, repeated three times.

Metabolomics assays were conducted on viable rhizome sections of *P. kingianum* plants. Three rhizomes per treatment were sliced, mixed, and analyzed in five biological replicates. Sample preparation and data analysis for metabolomics analyses were performed according to standard procedures at Wuhan Novogene Bioinformatics Technology Co., Ltd. (Wuhan, China). The raw mass spectrometry offline files were converted to the mzXML file format by the MSConvert tool in the Proteowizard software package (v3.0.8789) (Rasmussen et al., 2022). Peak detection, peak filtering, and peak alignment were processed using the R XCMS software package (Navarro-Reig et al., 2015) with parameters set with bw = 2, ppm = 15, peakwidth = c (5, 30), mzwid = 0.015, mzdiff = 0.01, method="centWave" to get the quantitative list of substances. Support vector regression correction based on QC samples was used to eliminate systematic errors. Substances with a coefficient of variation (CV) less than 30% in the QC samples were then retained for subsequent analysis (Want et al., 2013).

### 2.5 Transcriptomic analysis

Frozen samples were sent to Wuhan Novogene Bioinformatics Technology Co., Ltd. for transcriptome sequencing, with five biological replicates per treatment. RNA was extracted from the rootstocks of *P. kingianum* and purified for library construction, and Next-Generation Sequencing (NGS) was performed on an Illumina sequencing platform. Following sequencing, raw data were screened to detect sequencing error rates and GC content distribution to obtain clean reads for subsequent analysis.

### 2.6 Statistical analysis

Data were analyzed using Microsoft Excel v. 2010 (Microsoft Corporation, Redmond, WA, USA). Principal component analysis (PCA) was performed using the MetWare Cloud Platform (https://cloud.metware.cn/#/user/login). The R package (Metabo Analyst R) version 1.0.1 was used for graph construction.

## 3 Results

### 3.1 Effect of different concentrations of SeNPs treatments on selenium content in leaves and rhizomes of *P. kingianum*

**Figure 1:**
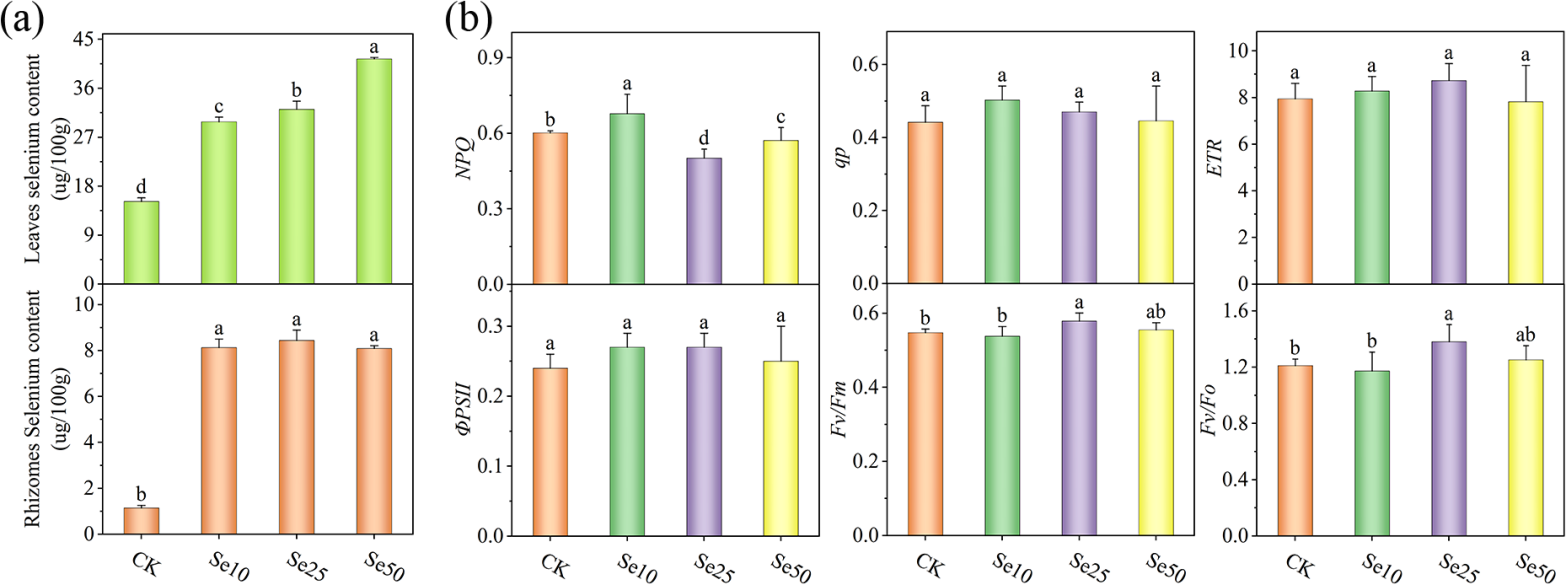
Effect of different SeNPs concentrations on **(A)** selenium accumulation and **(B)** chlorophyll fluorescence parameters. Vertical lines indicate the standard deviation of six replications. For the same parameter, different letters above the bars indicate significant differences (*P* < 0.05).

With the gradual increase in SeNPs concentration, the selenium content in *P. kingianum* rhizomes reached a certain level and then tended to stabilize, while the selenium content in the leaves increased in a gradient (**Figure 1A**). Among them, the selenium content of rhizomes was the highest in the Se25 treatment group, which was 7.38 times higher than that of CK, and the selenium content of leaves was the highest in the Se50 treatment group, which was 2.72 times higher than that of CK.

### 3.2 Effect of different concentrations of SeNPs treatments on chlorophyll fluorescence parameters of *P. kingianum* leaves

The chlorophyll fluorescence parameters of *P. kingianum* under SeNPs treatment were measured (**Figure 1B**), revealing that NPQ showed a trend of initially increasing and then decreasing, with the lowest NPQ observed in the Se25 treatment. Under Se25 treatment, there were no significant changes in *Φ*PSII, qp, and ETR compared to the CK. Fv/Fm reflects the maximum photochemical efficiency of the plant’s photosystem, while Fv/Fo is commonly used to characterize the efficiency of photosynthesis and the physiological state of the plant. In this study, both Fv/Fm and Fv/Fo significantly increased under the Se25 treatment, indicating that this selenium concentration can significantly enhance the photosynthetic potential and efficiency of *P. kingianum* leaves.

### 3.3 Effect of different concentrations of SeNPs treatments on the carbon to nitrogen ratio of *P. kingianum*

**Figure 2:**
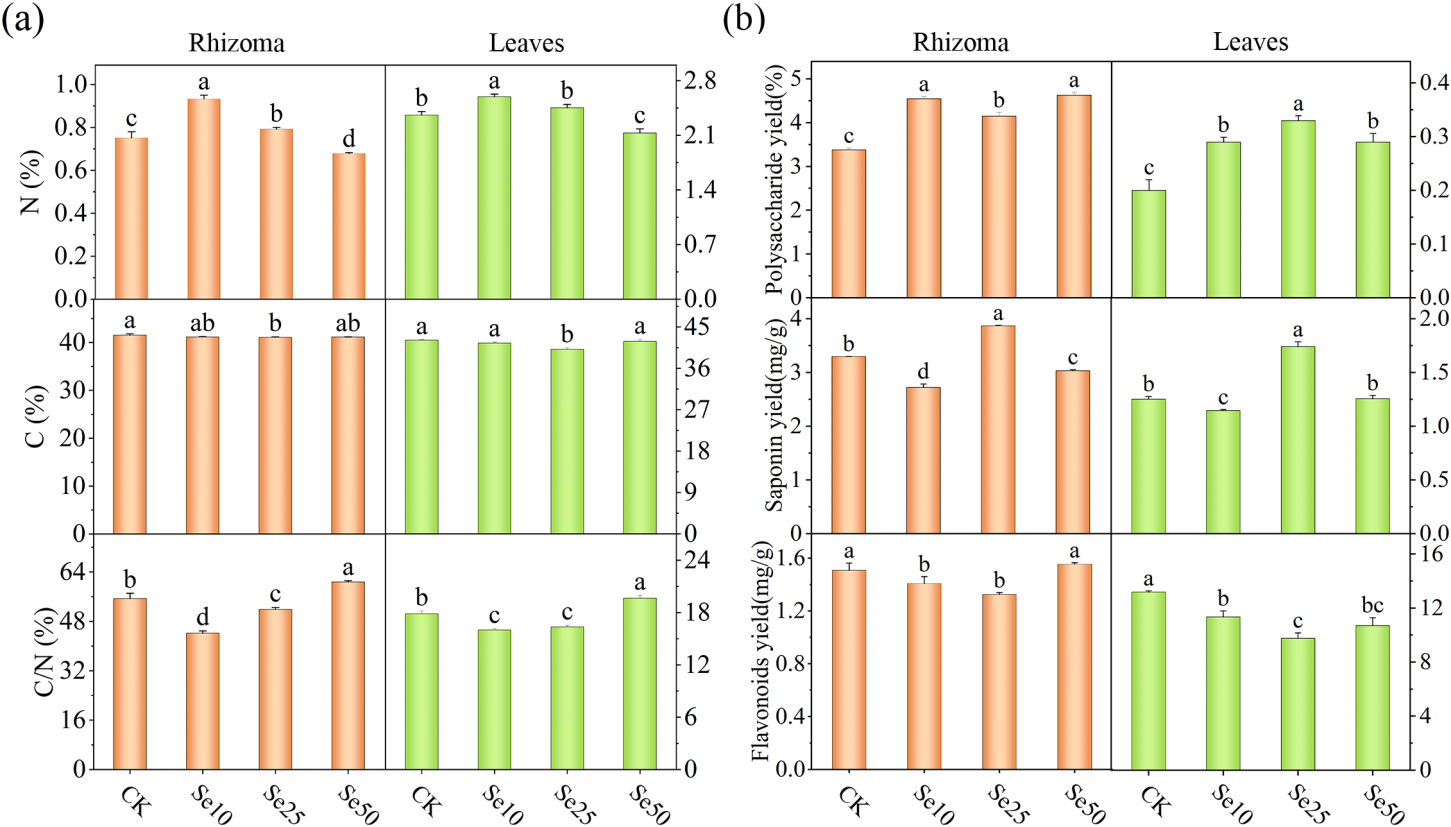
Effects of different SeNPs concentrations on **(A)** carbon and nitrogen content, and **(B)** major secondary metabolites. The vertical lines indicate the standard deviation of three replicates. For the same parameter, different letters above the bars indicate significant differences (*P* < 0.05).

As the concentration of SeNPs increased, we found that, compared to the CK, the nitrogen content in the rhizomes and leaves of *P. kingianum* under Se10 treatment significantly increased to 1.24 times and 1.07 times that of CK, respectively. Under Se25 treatment, the nitrogen content in the rhizomes and leaves of *P. kingianum* also significantly increased to 1.10 times and 1.04 times that of CK. However, under the Se50 treatment, the nitrogen content in both the rhizomes and leaves of *P. kingianum* significantly decreased compared to CK. Additionally, under Se25 treatment, the carbon content in both the rhizomes and leaves of *P. kingianum* significantly decreased, while no significant changes were observed in the other treatments compared to CK. Regarding the carbon-to-nitrogen ratio, both Se10 and Se25 treatments were significantly lower than CK, while the Se50 treatment was significantly higher (**Figure 2A**).

### 3.4 Effect of different concentrations of SeNPs treatments on the content of major secondary metabolites of *P. kingianum*

The content of major secondary metabolites in the leaves and rhizomes of *P. kingianum* varied significantly under different SeNPs concentrations. The polysaccharide content in *P. kingianum* treated with SeNPs was significantly higher compared to CK, indicating that SeNPs treatment can promote the accumulation of polysaccharides in the rhizomes and leaves. Under Se25 treatment, the saponin content in the rhizomes and leaves of *P. kingianum* significantly increased compared to CK, whereas the saponin content under other SeNPs treatments significantly decreased, suggesting that Se25 is the optimal concentration for saponin accumulation. Additionally, we found that the flavonoid content in the rhizomes and leaves of *P. kingianum* was lowest under Se25 treatment. In the rhizomes of *P. kingianum*, the flavonoid content in CK was 1.14 times that of the Se25 treatment. In the leaves of *P. kingianum*, the flavonoid content in CK was 1.35 times that of the Se25 treatment (**Figure 2B**).

### 3.5 Transcriptomic analysis

#### 3.5.1 Transcriptome sequencing and identification of differentially expressed genes

**Figure 3:**
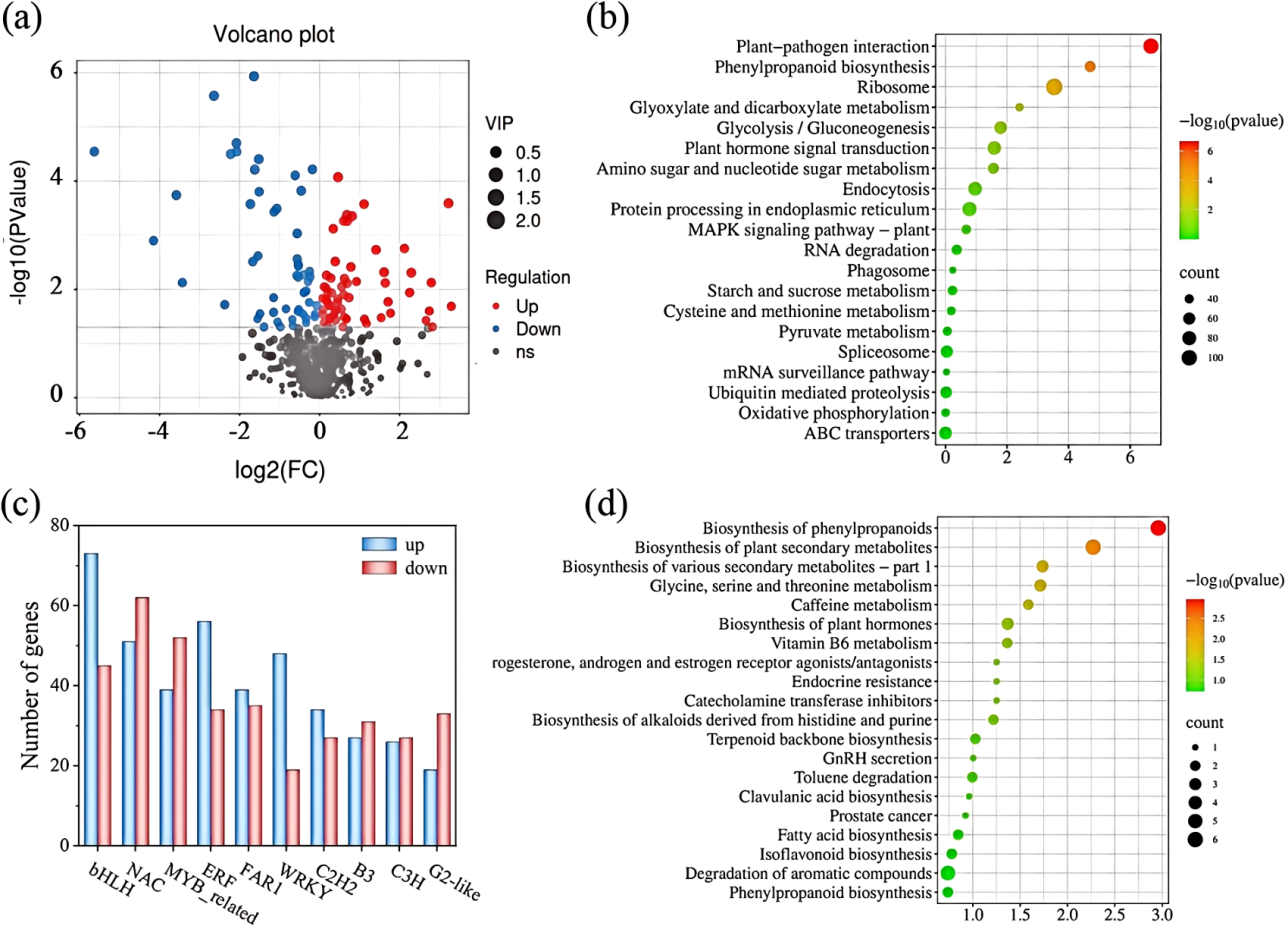
Transcription factor changes; GO enrichment analysis; KEGG enrichment analysis; and changes in differential metabolites. **(A)** Up- and down-regulation of different metabolites. **(B)** KEGG pathway enrichment analysis of differential genes. **(C)** Number of transcription factor differential genes. Upper calls are shown in red, and lower calls are shown in blue. **(D)** KEGG pathway enrichment analysis of different metabolites.

Due to the significant differences in various indicators between CK and Se25 treatments, transcriptome analysis was conducted on the rhizomes of these two treatments to determine the changes in gene expression in the rhizomes of the CK and Se25 groups. After removing low-quality reads, a total of 428,741,090 clean reads were obtained. The percentage of high-quality sequence reads in the sequenced reads ranged from 97.85% to 98.17%, indicating the high quality of transcriptome sequencing data (**Supplementary Table S1**). The percentage of fuzzy bases was less than 0.007%, and the Q30 was above 95% (**Supplementary Table S2**), indicating the applicability of the selected reference genome. **Supplementary Figure S1** shows the annotation information for each database. The correlation HCA of the samples showed that the correlation coefficient was greater than 0.6 for both treatment groups (**Supplementary Figure S2A**), and PCA analysis showed that there were significant differences in gene expression at different selenium treatment concentrations (**Supplementary Figure S2B**). In addition, |log2FoldChange| > 1, significance *P*-value < 0.05 screened for differentially expressed genes, as shown in **Supplementary Table S3**. 18,826 differentially expressed genes (8,604 up-regulated and 10,222 down-regulated) were identified in the comparison between CK and Se25 treatments. **Supplementary Table S4** lists the 20 genes with the highest variance. Transcription factors are a class of protein molecules that bind exclusively to specific sequences upstream of the 5’ end of a gene and form a transcription initiation complex with RNA polymerase II, which participates in the process of transcription initiation. The 10 transcription factors with the highest number of differential genes are listed in **Supplementary Table S5** and plotted in a bar graph (**Figure 3C**).

#### 3.5.2 GO analysis and KEGG pathway analysis

The Gene Ontology (GO) and Kyoto Encyclopedia of Genes and Genomes (KEGG) databases were further evaluated for the biological functions of the differential genes under CK and Se25 treatments. GO enrichment analyses were performed to categorize the enrichment of CK and Se25 treatments from the three ontologies (Biological Process (BP), Molecular Function (MF), and Cellular Component (CC)) (**Supplementary Figure S3**).

**Figure 3B** shows KEGG pathway enrichment analysis of all differential expression using bubble plots to investigate the effect of SeNPs treatment on the differentially enriched pathways. The downregulated differential genes in Se25 and CK were highly enriched in KEGG pathways such as flavonoid biosynthesis, pentose phosphate pathway, D-amino acid metabolism, glutathione metabolism, phenylalanine, tyrosine, and tryptophan biosynthesis. Under Se25 treatment, plant growth in the rhizomes of *P. kingianum* primarily promoted by regulating amino acid and organic acid metabolism.

### 3.6 Metabolomics analysis

#### 3.6.1 Quality control of metabolomic data

Targeted metabolomics (LC-MS) methods were employed to analyze the rhizomes of *P. kingianum*, aiming to identify a variety of plant metabolites associated with SeNPs treatment and to reveal the response mechanisms of the rhizomes under SeNPs treatment. This experiment used CK as the control group and the Se25 group as the SeNPs treatment group. A total of 670 metabolites were obtained from the samples, and all metabolites were categorized into 14 subclasses (**Supplementary Table S6**). The metabolite contents were normalized to hierarchical cluster analysis (HCA) graphical plotting (**Supplementary Figure S4**), followed by principal component analysis (PCA) graphical plotting (**Supplementary Figure S5**), which showed a clear separation between the two samples in both modes. It indicates that there are significant differences in metabolites within the rhizomes at different SeNPs concentrations. In this study, VIP values and *P*-value values (VIP > 1, *P*-value < 0.05) were combined to identify differential metabolites. There were 2351 differential metabolites (1011 up-regulated and 1340 down-regulated) in the primary metabolites in positive ion mode. In the negative ion mode, 2,952 differential primary metabolites were identified (886 up-regulated and 2,066 down-regulated).

#### 3.6.2 Metabolite types

A total of 670 secondary metabolites were detected in all samples, with 112 differential metabolites found between CK and Se25 (60 up-regulated and 52 down-regulated). Among them, terpenoids, phenylpropanoids, and flavonoids accounted for 14.2%, 7.1%, and 12.5%, respectively. Volcano plots were used to demonstrate the up- and down-regulation of differential metabolites (**Figure 3A**). A z-score plot was used to demonstrate the distribution of differential metabolites across groups (**Supplementary Figure S6**).

In a comparison of CK with Se25, increased accumulation of indole alkaloids, monolignols, sarcosine, and anthraquinone was found, whereas accumulation of saturated fatty acids, polyphenols, theophylline, and chalcone was reduced. In summary, changes in the content of different metabolites in the rhizomes of *P. kingianum* were correlated with SeNPs treatment, indicating that *P. kingianum* enhances its selenium tolerance by regulating the content of metabolites in the rhizomes.

#### 3.6.3 KEGG enrichment analysis

Through KEGG enrichment pathway analysis, we further identified the key metabolic pathways in the rhizomes of *P. kingianum* in response to SeNPs treatment. The analysis revealed that 76 metabolic pathways were involved in the differential expression of metabolites in *P. kingianum* plants under Se25 treatment (**Supplementary Table S7**). To directly analyze the differences in metabolic pathways between groups, the top 20 pathways with the highest enrichment in the control group were selected by enrichment analysis and topology analysis (**Figure 3D**). Pathways such as the biosynthesis of phenylpropanoids, the biosynthesis of plant secondary metabolites, the biosynthesis of isoflavonoids, the metabolism of glycine, serine, threonine metabolism, and the biosynthesis of alkaloids originating from the histidine and purine pathways were significantly enriched in a comparison of CK and Se25. These results are generally in agreement with the findings from transcriptomics.

### 3.7 Integrated analysis of transcriptomics and metabolomics

#### 3.7.1 Correlation between genes and metabolites

**Figure 4:**
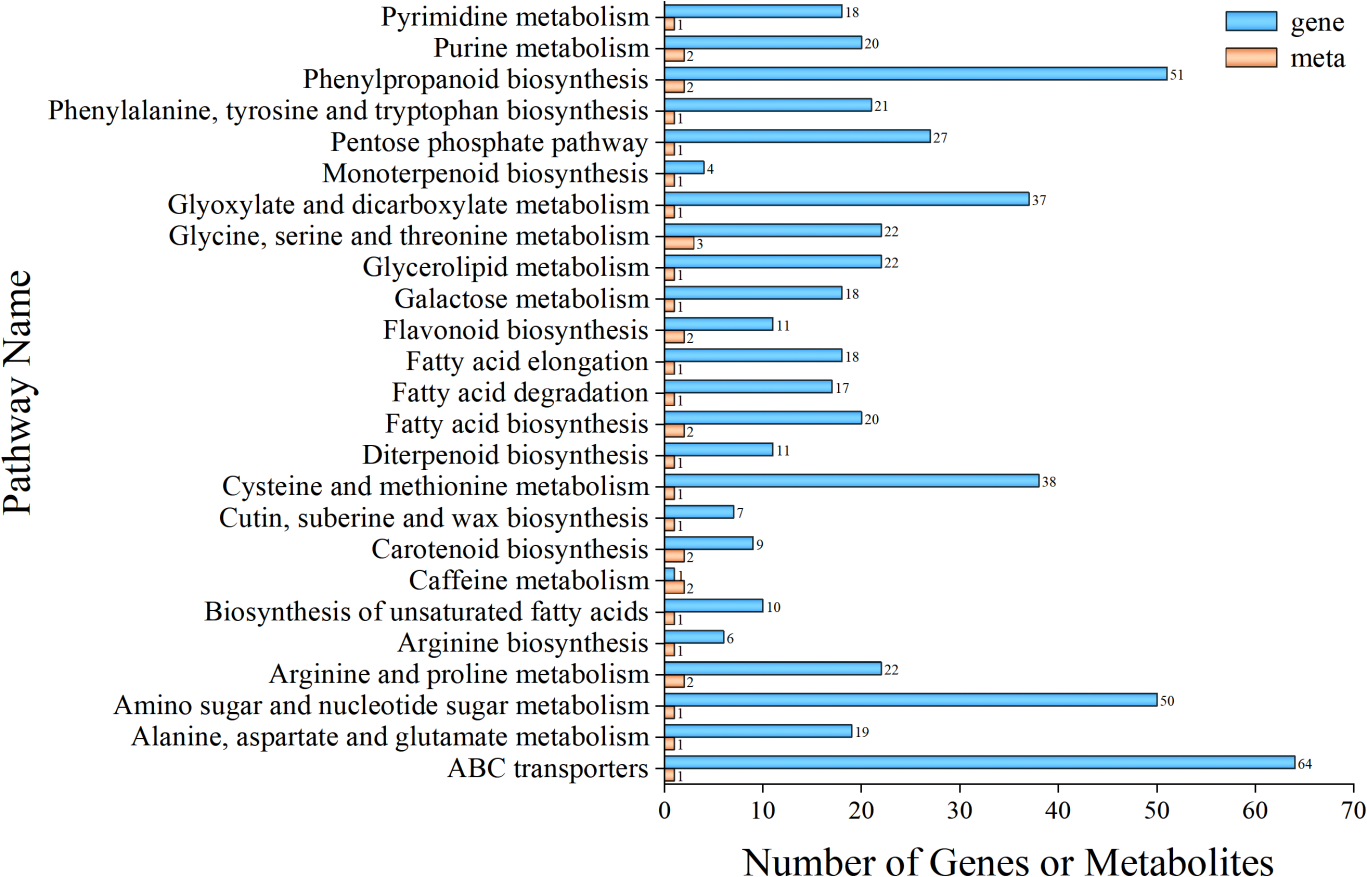
Correlation analysis between genes and metabolites. Orange represents differential metabolites enriched in the metabolome, and blue represents differential genes enriched in the transcriptome.

To investigate the relationship between differential genes and differential metabolites in *P. kingianum* rhizomes under SeNPs treatment, we performed co-expression network analysis of differential genes and differential metabolites (Pearson’s correlation coefficient > 0.8, *P*-value < 0.05). KEGG enrichment analysis revealed that 32 KEGG pathways were shared by both the metabolome and transcriptome when comparing CK and Se25 (**Supplementary Table S8**). For shared KEGG pathways, the 25 pathways with the highest *P*-values were shown (**Figure 4**).

#### 3.7.2 Analysis of key pathways for major secondary metabolites

**Figure 5:**
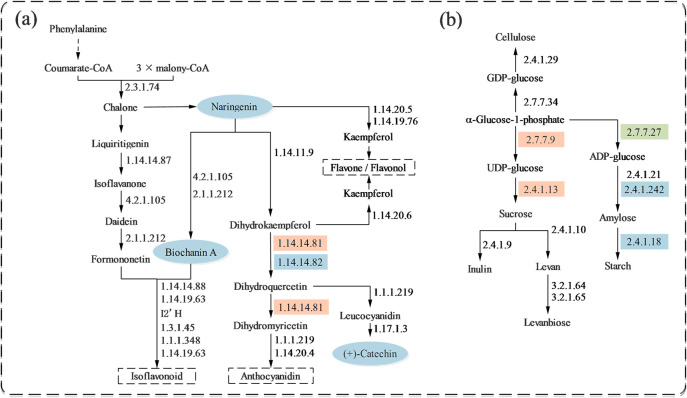
Changes in metabolic pathways of major secondary metabolites in the rhizomes of *P. kingianum* after 40 days of treatment with different concentrations of selenium. **(A)** Differential metabolites and genes in the flavonoid metabolic pathway under SeNPs treatment (CHS: chalcone synthase; IFS: isoflavonoid synthase; HID: 2-hydroxyisoflavanone dehydratase; HMM1: 2,7,4’-trihydroxyisoflavanone 4’-O-methyltransferase; CYP81E9: isoflavone 3’-hydroxylase; PBS: pseudobaptigenin synthase; IFR: isoflavone reductase; VR: vestitone reductase; F3H: flavanone 3-dioxygenase; F3’5’H: flavanoid 3’,5’-hydroxylase; CYP75B1: flavonoid 3’-monooxygenase; DFR: dihydroflavonol 4-reductase; ANS: anthocyanidin synthase; FNSI: flavone synthase I; CYP93B16: flavone synthase II; FLS: flavonol synthase; LAR: leucoanthocyanidin reductase). **(B)** Differential metabolites and genes in the polysaccharide metabolic pathway under SeNPs treatment (UGPase: UDP glucose pyrophosphorylase; CesA: cellulose synthase; GPT: glucose-1-phosphate guanylyltransferase; SUS: sucrose synthase; ISU: inulosucrase; SFT: sucrose 6-fructosyltransferase; 6-FFH: beta-2,6-fructan-6-levanbiohydrolase; INU: 2,6-beta-D-fructan fructanohydrolase; GCK: glucose-1-phosphate adenylyltransferase; AGS: ADP-glucose starch synthase; GBSSI: NDP-glucose-starch glucosyltransferase; BE: 1,4-alpha-glucan branching enzyme). Blue ovals represent metabolites with reduced content. Orange rectangles represent enzymes that were up-regulated by the differential gene, blue rectangles represent enzymes that were down-regulated by the differential gene, and green rectangles represent enzymes that underwent both down- and up-regulation by the differential gene.

By integrating transcriptome and metabolome analyses, we found that polysaccharide differential genes were mainly enriched in the starch and sucrose metabolism pathways. In contrast, differential genes and metabolites related to flavonoid compounds were mainly enriched in the flavonoid biosynthesis and isoflavonoid biosynthesis pathways (**Figure 5**).

In the metabolomic data, there were 84 flavonoid compounds, among which 14 showed differential changes (5 upregulated and 9 downregulated) (**Supplementary Table S9**). The Se25 treatment reduced the accumulation of naringenin, which in turn affected the accumulation of biochanin A in the isoflavonoid biosynthesis pathway and (+)-catechin in the flavonoid biosynthesis pathway. In the biosynthesis of phenylpropanoid pathways, the Se25 treatment was found to decrease the accumulation of naringenin, (+)-catechin, and biochanin A. In the starch and sucrose metabolism pathway, we observed an increase in enzyme activity regulating sucrose synthesis and a decrease in enzyme activity regulating starch synthesis, while the enzyme activity regulating cellulose synthesis did not show significant changes.

## 4 Discussion

### 4.1 Effect of SeNPs on chlorophyll fluorescence parameters in *P. kingianum*

In this study, multi-omics analysis was used to explore the response mechanisms of *P. kingianum* plants under different SeNPs concentrations. The polysaccharide and saponin contents and flavonoid contents were significantly higher and lower in *P. kingianum* rhizomes and leaves under Se25 treatment compared to CK. The chlorophyll fluorescence parameters such as Fo, Fm, Fv, Fv/Fo, and Fv/Fm are important parameters of primary PSII photochemistry and can serve as reliable diagnostic indicators for environmental damage (Zhu et al., 2010). When plants are subjected to stressful environmental conditions, such as nitrogen and phosphorus deficiency (Rattan et al., 2012) lead poisoning (Li et al., 2012), and iron deficiency (Donnini et al., 2013), an increase in Fo or a decrease in Fm, Fv/Fo, and Fv/Fm can be detected. In this study, the Se25 treatment significantly reduced the NPQ value of *P. kingianum* leaves while markedly increasing Fv/Fm and Fv/Fo values. This suggests that Se25 might have exerted a certain level of stress on *P. kingianum*, prompting them to enhance their adaptability to environmental stress and thereby improving photosynthetic efficiency. Our result is similar to the results observed in *Landoltia punctata* (Zhong et al., 2016).

### 4.2 Effects of SeNPs on major secondary metabolites in *P. kingianum*

The rhizome metabolome revealed that the Se25 treatment decreased concentration of three flavonoid compounds, naringenin, biochanin A, and (+)-catechin, which are involved in the synthesis of flavonoid compounds in the flavonoid biosynthesis and isoflavonoid biosynthesis pathways. These compounds provide precursor molecules and the phenylpropanoid skeleton for the synthesis of flavonoid compounds in the rhizomes. Additionally, the synthesis of Biochanin A can provide an isoflavone skeleton for the synthesis of flavonoids, thus providing more possibilities for flavonoid diversity. Even under salt stress conditions, selenium can increase the content of flavonoid compounds in plants (Ghanbari et al., 2023). The decrease in flavonoid compound content observed in the preliminary stages of this study may lead to a reduction in the antioxidant activity and free radical scavenging ability of the plants. In the present study, Se25 treatment up-regulated 6-phosphogluconic acid dehydrogenase (G6PD)-related genes in the pentose phosphate pathway, which promoted the conversion of 6-phosphogluconic acid lactone and increased the content of 6-phosphogluconic acid, which may be related to the accumulation of sugars in the rhizomes. In the starch and sucrose metabolism pathway, Se25 treatment up-regulated the SUS and UGPase enzyme genes associated with sucrose synthesis, which provided the energy and carbon skeleton for the growth of the rhizomes of *P. kingianum*, as well as the basis for the accumulation of polysaccharide content (**Figure 5**). Previous studies have shown that SUS can synthesize sucrose reversibly, and SPS catalyzes the conversion of uridine diphosphate glucose and fructose-6-phosphate to sucrose-6-phosphate, which is then irreversibly converted to sucrose by SPP (Liao et al., 2022). UGPase is one of the most important regulatory enzymes for sucrose and starch metabolism and secondary cell wall synthesis. It catalyzes the reversible formation of glucose 1-phosphate (Glc-1-P) and uridine triphosphate (UTP) into uridine diphosphate glucose (UDP-Glc) (Yousaf et al., 2021). In addition, enzyme genes such as amylase (Amy), sucrose phosphate synthase (SPS), and ADP-glucose pyrophosphorylase (AGPase) in the starch and sucrose metabolism pathways were also upregulated. AGPase has been found to convert ATP and Glc-1-p to ADP-glucose and diphosphate in starch biosynthesis and is a key enzyme in starch synthesis (Oiestad et al., 2019; Sun et al., 2020; Yu et al., 2019). Amy hydrolyzes starch to glucose, which provides raw material for sucrose synthesis (Li et al., 2022). The upregulation of genes related to SUS, SPS, and AGPase also indicates that plants can resist the adverse effects of selenium on rhizomes by accumulating large amounts of sugars in the root system (Liu et al., 2024). Sugars can be used as small-molecule signaling substances against abiotic stresses (Siddiqui et al., 2020), and soluble sugars in rhizomes are not only energy storage substances and osmoregulators in plants, but also play a key role as signaling substances under drought and heavy-metal stresses (Wei et al., 2019; Meng et al., 2022). Analyzing the relevant pathways of saponin synthesis under Se25 treatment, we found that 15 saponin-like metabolites were differentially changed (9 up-regulated and 6 down-regulated) (**Supplementary Table S10**). Whereas the accumulation of mevalonate-5P in the terpenoid backbone biosynthesis pathway, as well as protein arginine deiminase (PAD), alcohol dehydrogenase (ADH), pyruvate kinase (PK), and glutamine synthetase (GS), and other related genes, were up-regulated. The genes related to (-)-germacrene D synthase in the Sesquiterpenoid and triterpenoid biosynthesis pathways were also up-regulated. Combined with the results of the preliminary assay, the saponin content in both leaves and rhizomes increased. This is similar to previous research findings: foliar application of SeNPs can increase the saponin content in the roots of *Panax notoginseng* (Dong Q. et al., 2022); application of selenium nanoparticles coated with polyethylene glycol chitosan on ginseng can significantly upregulate the biosynthesis genes of ginsenosides (PgHMGR, PgSS, PgSE, and PgDDS) (Abid et al., 2021).

### 4.3 Association analysis of major secondary metabolites

In summary, we believe that there is a widespread connection between polysaccharides, flavonoids, and saponin in plants, leading to a decrease in flavonoid content when the content of polysaccharides and saponin increases. For example, in the terpenoid backbone biosynthesis pathway, we found that the content of geranylgeranyl diphosphate (GGPP) decreased, which can indirectly lead to an increase in the content of zeaxanthin in the carotenoid biosynthesis, and GGPP is also one of the important substrates for saponin synthesis. Therefore, we believe that the synthesis of saponins consumes some of the compounds related to flavonoid synthesis, which leads to a decrease in the accumulation of flavonoids. In previous studies, it was found that low-temperature treatment of jujube plants could promote the accumulation of ferulic acid and flavonoids, but inhibit polysaccharide biosynthesis (Dong H. et al., 2022). Thus, in this study, the content of saponin and flavonoids in the rhizomes of *P. kingianum* showed a negative correlation under Se25 treatment, and GGPP is an important factor causing this negative correlation. Sugars play an important role in the synthesis of saponin in plants. It has been discovered that the C-20 of sapogenin aglycone has an S-configuration, and the sugar units are D-glucose, L-rhamnose, and D-xylose, which are connected to the aglycone through 3β-OH (PPD) or 6α-OH (PPT) (Shen et al., 2016). D-glucose, L-rhamnose, and D-xylose are monosaccharides, while polysaccharides are polymeric carbohydrate macromolecules composed of sugar chains linked by glycosidic bonds, consisting of more than 10 monosaccharides. Thus, the accumulation of saponin and polysaccharide contents in the rhizomes of *P. kingianum* was positively correlated under Se25 treatment. It has been found that an increase in photosynthetic rate can enhance the activity of sucrose phosphate synthase, thereby improving the efficiency of sucrose transfer (Li et al., 2021). The Fv/Fm and Fv/Fo measured in this study increased significantly, as did the UGPase and SUS activities associated with sucrose synthesis. This suggests that the increase in photosynthetic potential and photosynthetic efficiency helps to catalyze UGPase and SUS activities, which indirectly affect polysaccharide synthesis in *P. kingianum* plants.

### 4.4 Effects of SeNPs on the major carbon and nitrogen metabolism of *P. kingianum*

Carbon and nitrogen metabolism are closely interconnected. Carbon metabolism provides the energy and carbon sources required for nitrogen metabolism, while the enzymes and photosynthetic pigments needed for carbon metabolism depend on nitrogen metabolism. This interplay has a significant impact on plant growth (Duan et al., 2023; Cui et al., 2019). In addition, nitrogen metabolism has been found to be able to influence the accumulation of plant secondary metabolites. For example, the nitrogen metabolism of *Andrographis paniculata* has been studied, and it was found that the reduction of nitrogen metabolism reduced Andrographis paniculata carbohydrate consumption and synergistically promoted plant growth and *Andrographis paniculata* lactone biosynthesis (Zhong et al., 2021). This study found that the flavonoid content in *P. kingianum* is positively correlated with the carbon-to-nitrogen ratio, which aligns with findings from research on *Broussonetia papyrifera* (Deng et al., 2022). Additionally, under Se25 treatment, the carbon-to-nitrogen ratio was negatively correlated with the content of polysaccharides and saponins. In previous studies, glutamate has been used as a nitrogen source in the biosynthesis of nitrogen compounds (Qunfeng et al., 2017). Cysteine is the first organic product of sulfur production (Li et al., 2020), cysteine synthase is the last enzyme of the sulfate assimilation pathway, and its precursor O-acetylserine is derived from the carbon and nitrogen assimilation pathways (Koprivova et al., 2000), and ornithine and arginine are used to store excess organic nitrogen in plants (Howarth et al., 2008). This demonstrates the ability of SeNPs treatment to affect the metabolic pathways of various amino acids and thus the amount of nitrogen accumulation. Under Se25 treatment, the nitrogen content increased, and photosynthesis was also enhanced, similar to findings from previous studies on plants such as sugarcane and *Panax notoginseng* (Denis et al., 2018; Zhang et al., 2020). The significant decrease in carbon content may be due to the consumption of carbon through plant respiration, growth, and metabolism exceeding the amount fixed by photosynthesis (Vanlerberghea et al., 2020).

## 5 Conclusion

This study used multi-omics analysis to investigate the response mechanisms of *P. kingianum* plants to different SeNPs concentrations. The findings revealed that under the Se25 treatment, *P. kingianum* plants’ photosynthetic electron transport rate significantly increased while the carbon-to-nitrogen ratio decreased. The content of major secondary metabolites, including *P. kingianum* polysaccharides and saponins, increased significantly, while the content of flavonoids decreased. Transcriptome and metabolome analyses indicated that SeNPs primarily influenced pathways such as starch and sucrose metabolism, flavonoid biosynthesis, isoflavonoid biosynthesis, phenylpropanoid biosynthesis, and sesquiterpenoid and triterpenoid biosynthesis. Se25 treatment upregulated genes such as SPS, UGPase, AGPase, UTP, and SUS, thereby enhancing the starch and sucrose metabolism pathway and increasing polysaccharide content to mitigate adverse effects of SeNPs on the roots. Moreover, Se25 treatment upregulated genes, including PAD, ADH, PK, and GS, regulating the sesquiterpenoid and triterpenoid biosynthesis pathways and boosting saponin content. However, we also found that GGPP may be a critical factor in a negative correlation between saponin and flavonoid content in *P. kingianum*. The accumulation of monosaccharides simultaneously influenced the accumulation of polysaccharides and saponins, leading to a positive correlation between polysaccharide and saponin levels in *P. kingianum* plants. In summary, our results provide new insights into the molecular pathways underlying *P. kingianum* plants’ response to varying SeNPs supply levels and offer guidance for the safe production of selenium-enriched medicinal materials while reducing environmental pollution.

## Data Availability

The original contributions presented in the study are included in the article/**Supplementary Material**. Further inquiries can be directed to the corresponding authors.

## References

[B1] Abid S. Kaliraj L. Rahimi S. Kim Y. J. Yang D. C. Kang S. C.. (2021). Synthesis and characterization of glycol chitosan coated selenium nanoparticles acts synergistically to alleviate oxidative stress and increase ginsenoside content in. Panax ginseng. Carbohydr. Polymers 267, 118195. doi: 10.1016/j.carbpol.2021.118195 34119162

[B2] Angamuthu A. Venkidusamy K. Muthuswami R. R. (2019). Synthesis and characterization of nano-selenium and its antibacterial response on some important human pathogens. Curr. Science: A Fortnightly J. Res. 116, 118195. doi: 10.18520/cs/v116/i2/285-290

[B3] Bhadwal S. Sharma S. (2022). Selenium alleviates physiological traits, nutrient uptake and nitrogen metabolism in rice under arsenate stress. Environ. Sci. Pollut. Res. 29, 70862–70881. doi: 10.1007/s11356-022-20762-5 35589895

[B4] Borbely P. Molnar A. Valyon E. Ordog A. Horvath-Boros K. Csupor D.. (2021). The effect of foliar selenium (Se) treatment on growth, photosynthesis, and oxidative-nitrosative signalling of *stevia rebaudiana* leaves. Antioxidants 10, 72. doi: 10.3390/antiox10010072 33429850 PMC7826996

[B5] Cui G. Zhang Y. Zhang W. Lang D. Zhang X. (2019). Response of carbon and nitrogen metabolism and secondary metabolites to drought stress and salt stress in plants. J. Plant Biol. 62, 387–399. doi: 10.1007/s12374-019-0257-1

[B6] Deng R. Lan Z. Shang X. Fang S. (2022). Effects of Biochar Application Pyrolyzed at Different Temperatures on Soil Properties, Growth and Leaf Secondary Metabolite Accumulation in. Cyclocarya paliurus. Forests 13, 1572. doi: 10.3390/f13101572

[B7] Denis B. Marcelo M. Lucia M. (2018). Nitrogen supply influences photosynthesis establishment along the sugarcane leaf. Sci. Rep. 8, 2327. doi: 10.1038/s41598-018-20653-1 29396510 PMC5797232

[B8] Djanaguiraman M. Belliraj N. Bossmann S. H. Prasad P. V. (2018). High-temperature stress alleviation by selenium nanoparticle treatment in grain sorghum. ACS Omega 3, 2479–2491. doi: 10.1021/acsomega.7b01934 31458542 PMC6641442

[B9] Dong H. Li M. Jin L. Xie X. Li M. Wei J. (2022). Cool temperature enhances growth, ferulic acid and flavonoid biosynthesis while inhibiting polysaccharide biosynthesis in angelica sinensis. Molecules 27, 320. doi: 10.3390/molecules27010320 35011549 PMC8746531

[B10] Dong Q. Yan S. Li D. Zhou C. Tian S. Wang Y.. (2022). Feeding foliar nano-selenium biofortified panax notoginseng could reduce the occurrence of glycolipid metabolism disorder in mice caused by high-fat diets. Front. Nutr. 9, 973027. doi: 10.3389/fnut.2022.973027 PMC9450130 36091251

[B11] Donnini S. Guidi L. Degl'Innocenti E. Zocchi G. (2013). Image changes in chlorophyll fluorescence of cucumber leaves in response to iron deficiency and resupply. J. Plant Nutr. Soil Sci. 176, 734–742. doi: 10.1002/jpln.201200479

[B12] Duan Y. Yang H. Yang H. Wu Y. Fan S. Wu W.. (2023). Integrative physiological, metabolomic and transcriptomic analysis reveals nitrogen preference and carbon and nitrogen metabolism in blackberry plants. J. Plant Physiol. 280, 153888. doi: 10.1016/j.jplph.2022.153888 36577314

[B13] DuBois M. Gilles K. Hamilton J. Rebers P. Smith F. (1956). Colorimetric method for determination of sugars and related substances. Anal. Chem. 28, 350–356. doi: 10.1021/ac60111a017

[B14] Ghanbari F. Bag-Nazari M. Azizi A. (2023). Exogenous application of selenium and nano-selenium alleviates salt stress and improves secondary metabolites in lemon verbena under salinity stress. Sci. Rep. 13, 5352. doi: 10.1038/s41598-023-32436-4 37005438 PMC10067816

[B15] Hadrup N. Ravn-Haren G. (2021). Absorption, distribution, metabolism and excretion (ADME) of oral selenium from organic and inorganic sources: A review. J. Trace Elements Med. Biol. 67, 126801. doi: 10.1016/j.jtemb.2021.126801 34091241

[B16] Hajiboland R. Rahmat S. Zeinalzadeh N. Farsad-Akhtar N. Hosseinpour-Feizi M. A. (2019). Senescence is delayed by selenium in oilseed rape plants. J. Trace Elements Med. Biol. 55, 96–106. doi: 10.1016/j.jtemb.2019.06.005 31345373

[B17] Hajlaoui F. Hajlaoui H. Krouma A. (2023). Physio-Biochemical Response to Exogenous Selenium Application of Tomatoes (*Solanum lycopersicum* L.) Cultivated in the Field under Saline Irrigation. Russian J. Plant Physiol. 70, 142. doi: 10.1134/s1021443723601593

[B18] Hartikainen H. (2005). Biogeochemistry of selenium and its impact on food chain quality and human health. J. Trace Elements Med. Biol. 18, 309–318. doi: 10.1016/j.jtemb.2005.02.009 16028492

[B19] Hawrylak-Nowak B. Matraszek R. Pogorzelec M. (2015). The dual effects of two inorganic selenium forms on the growth, selected physiological parameters and macronutrients accumulation in cucumber plants. Acta Physiologiae Plantarum 37, 41. doi: 10.1007/s11738-015-1788-9

[B20] Howarth J. R. Parmar S. Jones J. Shepherd C. E. Corol D. Galster A. M.. (2008). Co-ordinated expression of amino acid metabolism in response to N and S deficiency during wheat grain filling. J. Exp. Bot. 59, 3675–3689. doi: 10.1093/jxb/ern218 18791197 PMC2561146

[B21] Khan H. Saeed M. Muhammad N. Perviz S. (2013). Phytochemical analysis, antibacterial, and antifungal assessment of aerial parts of Polygonatum verticillatum. Toxicol. Ind. Health 32, 841–874. doi: 10.1177/0748233713512362 24311628

[B22] Koprivova A. Suter M. den Camp R. O. Brunold C. Kopriva S. (2000). Regulation of sulfate assimilation by nitrogen in Arabidopsis. Plant Physiol. 122, 737–746. doi: 10.1104/pp.122.3.737 10712537 PMC58909

[B23] Li X. Bu N. Li Y. Ma L. Xin S. Zhang L. (2012). Growth, photosynthesis and antioxidant responses of endophyte infected and non-infected rice under lead stress conditions. J. Hazardous Materials 213-214, 55–61. doi: 10.1016/j.jhazmat.2012.01.052 22356744

[B24] Li Q. Gao Y. Yang A. (2020). Sulfur homeostasis in plants. Int. J. Mol. Sci. 21, 8926. doi: 10.3390/ijms21238926 33255536 PMC7727837

[B25] Li Y. Lv Y. Lian M. Peng F. Xiao Y. (2021). Effects of combined glycine and urea fertilizer application on the photosynthesis, sucrose metabolism, and fruit development of peach. Scientia Hortic. 289, 110504. doi: 10.1016/j.scienta.2021.110504

[B26] Li R. Zhang H. Pan S. Zhu M. Zheng Y. (2022). Preparation of slowly digested corn starch using branching enzyme and immobilized α-amylase. ACS omega 7, 17632–17640. doi: 10.1021/acsomega.2c00462 35664616 PMC9161404

[B27] Liao G. Li Y. Wang H. Liu Q. Zhong M. Jia D.. (2022). Genome-wide identification and expression profiling analysis of sucrose synthase (SUS) and sucrose phosphate synthase (SPS) genes family in *Actinidia chinensis* and A. eriantha. BMC Plant Biol. 22, 1–15. doi: 10.1186/s12870-022-03603-y 35468728 PMC9040251

[B28] Liu C. Zhou G. Qin H. Guan Y. Wang T. Ni W.. (2024). Metabolomics combined with physiology and transcriptomics reveal key metabolic pathway responses in apple plants exposure to different selenium concentrations. J. Hazardous Materials 464, 132953. doi: 10.1016/j.jhazmat.2023.132953 37952334

[B29] Lu J. Wang Y. Yan H. Lin P. Gu W. Yu J. (2016). Antidiabetic effect of total saponins from *Polygonatum kingianum* in streptozotocin-induced daibetic rats. J. Ethnopharmacology 179, 291–300. doi: 10.1016/j.jep.2015.12.057 26743227

[B30] Mao J. Pop V. J. Bath S. C. Vader H. L. Redman C. W. G. Rayman M. P. (2016). Effect of low-dose selenium on thyroid autoimmunity and thyroid function in UK pregnant women with mild-to-moderate iodine deficiency. Eur. J. Nutr. 55, 55–61. doi: 10.1007/s00394-014-0822-9 PMC4737786 25524327

[B31] Meng L. Yang Y. Ma Z. Jiang J. Zhang X. Chen Z.. (2022). Integrated physiological, transcriptomic and metabolomic analysis of the response of *Trifolium pratense* L. @ to Pb toxicity. J. Hazardous Materials 436, 129128. doi: 10.1016/j.jhazmat.2022.129128 35594664

[B32] Mittler R. (2017). ROS are good. Trends Plant Sci. 22, 11–19. doi: 10.1016/j.tplants.2016.08.002 27666517

[B33] Navarro-Reig M. Jaumot J. Garcia-Reiriz A. Tauler R. (2015). Evaluation of changes induced in rice metabolome by Cd and Cu exposure using LC-MS with XCMS and MCR-ALS data analysis strategies. Anal. Bioanal Chem. 407, 8835–8847. doi: 10.1007/s00216-015-9042-2 26403240

[B34] Nothstein A. K. Eiche E. Riemann M. Nick P. Winkel L. H. E. Gttlicher J.. (2017). Tracking se assimilation and speciation through the rice plant -nutrient competition, toxicity and distribution. PloS One 11, e152081. doi: 10.1371/journal.pone.0152081 PMC4846085 27116220

[B35] Oiestad A. J. Martin J. M. Giroux M. J. (2019). Yield increases resulting from AGPase overexpression in rice are reliant on plant nutritional status. Plant Growth Regul. 89, 179–190. doi: 10.1007/s10725-019-00525-y

[B36] Puccinelli M. Malorgio F. Pezzarossa B. (2017). Selenium enrichment of horticultural crops. Molecules 22, 933. doi: 10.3390/molecules22060933 28587216 PMC6152644

[B37] Qunfeng Z. Meiya L. Jianyun R. (2017). Integrated transcriptome and metabolic analyses reveals novel insights into free amino acid metabolism in *huangjinya* tea cultivar. Front. Plant Sci. 8. doi: 10.3389/fpls.2017.00291 PMC5337497 28321230

[B38] Rasmussen J. A. Villumsen K. R. Ernst M. Hansen M. Forberg T. Gopalakrishnan S.. (2022). A multi-omics approach unravels metagenomic and metabolic alterations of a probiotic and synbiotic additive in rainbow trout (*Oncorhynchus mykiss*). Microbiome 10, 21. doi: 10.1186/s40168-021-01221-8 35094708 PMC8802455

[B39] Rattan K. J. Taylor W. D. Smith R. E. H. (2012). Nutrient status of phytoplankton across a trophic gradient in Lake Erie: evidence from new fluorescence methods. Can. J. Fisheries Aquat. Sci. 69, 94–111. doi: 10.1139/f2011-135

[B40] Razaghi A. Poorebrahim M. Sarhan D. Bjornstedt M. (2021). Selenium stimulates the antitumour immunity: Insights to future research. Eur. J. Cancer 155, 256–267. doi: 10.1016/j.ejca.2021.07.013 34392068

[B41] Rofiqah U. Fakhrurozi M. Hafidhuddin M. H. (2022). Extraction of flavonoid compound of bitter melon (*Momordica charantia* L.) fruit and leaves using the soxhlet method in different types of solvent. Materials Sci. Forum 1051, 58–63. doi: 10.4028/www.scientific.net/MSF.1051.58

[B42] Shen R. Cao X. Laval S. Sun J. Yu B. (2016). Synthesis of ocotillol-type ginsenosides. J. Organic Chem. 81, 10279–10294. doi: 10.1021/acs.joc.6b01265 27400182

[B43] Siddiqui H. Sami F. Hayat S. (2020). Glucose: Sweet or bitter effects in plants-a review on current and future perspective. Carbohydr. Res. 487, 107884. doi: 10.1016/j.carres.2019.107884 31811968

[B44] Sun H. Li J. Song H. Yang D. Deng X. Liu J.. (2020). Comprehensive analysis of AGPase genes uncovers their potential roles in starch biosynthesis in lotus seed. BMC Plant Biol. 20, 457. doi: 10.1186/s12870-020-02666-z 33023477 PMC7541243

[B45] Vanlerberghea G. C. Dahalb K. Albera N. A. Chadeea A. (2020). Photosynthesis, respiration and growth: A carbon and energy balancing act for alternative oxidase. Mitochondrion 52, 197–211. doi: 10.1016/j.mito.2020.04.001 32278748

[B46] Wang L. Zhang J. Yu H. (2007). Elemental selenium at nano size possesses lower toxicity without compromising the fundamental effect on selenoenzymes: comparison with selenomethionine in mice. Free Radic. Biol. Med. 42, 1524–1533. doi: 10.1016/j.freeradbiomed.2007.02.013 17448899

[B47] Wang M. Ali F. Qi M. Peng Q. Wang M. Banuelos G. S.. (2021). Insights into uptake, accumulation, and subcellular distribution of selenium among eight wheat (*Triticum aestivum* L.) cultivars supplied with selenite and selenate. Ecotoxicol Environ. Saf. 207, 111544. doi: 10.1016/j.ecoenv.2020.111544 33254403

[B48] Want E. J. Masson P. Michopoulos F. Wilson I. D. Theodoridis G. Plumb R. S.. (2013). Global metabolic profiling of animal and human tissues via UPLC-MS. Nat. Protoc. 8, 17–32. doi: 10.1038/nprot.2012.135 23222455

[B49] Wei T. Wang Y. Xie Z. Guo D. Chen C. Fan Q.. (2019). Enhanced ROS scavenging and sugar accumulation contribute to drought tolerance of naturally occurring autotetraploids in Poncirus trifoliata. Plant Biotechnol. J. 17, 1394–1407. doi: 10.1111/pbi.13064 30578709 PMC6576089

[B50] Yousaf M. F. Demirel U. Naeem M. Alkan M. E. (2021). Association mapping reveals novel genomic regions controlling some root and stolon traits in tetraploid potato (*Solanum tuberosum* L.). 3 Biotech. 11, 174. doi: 10.1134/s1021443722602749 PMC7973339 33927965

[B51] Yu G. Lv Y. Shen L. Wang Y. Qing Y. Wu N.. (2019). The proteomic analysis of maize endosperm protein enriched by phos-tagtm reveals the phosphorylation of brittle-2 subunit of ADP-Glc pyrophosphorylase in starch biosynthesis process. Int. J. Mol. Sci. 20, 986. doi: 10.3390/ijms20040986 30813492 PMC6412418

[B52] Zhang L. Chang Q. Wang Y. Liu H. Song Z. (2019). Optimization of extraction process of total polysaccharides, saponins and flavonoids in stem of huangjing (*Polygonatum sibiricum*) by response surface method. Guiding J. Traditional Chin. Med. Pharm. 25, 64–67. doi: 10.13862/j.cnki.cn43-1446/r.2019.08.018

[B53] Zhang J. Cun Z. Chen J. (2020). Photosynthetic performance and photosynthesis-related gene expression coordinated in a shade-tolerant species *Panax notoginseng* under nitrogen regimes. BMC Plant Biol. 20, 273. doi: 10.1186/s12870-020-02434-z 32593292 PMC7321538

[B54] Zhao P. Wu Z. Zheng Y. Shen J. Zhu Y. Chen Q.. (2023). Selenite affected photosynthesis of *Oryza sativa* L. exposed to antimonite: Electron transfer, carbon fixation, pigment synthesis via a combined analysis of physiology and transcriptome. Plant Physiol. Biochem. 201, 107904. doi: 10.1016/j.plaphy.2023.107904 37506651

[B55] Zhong C. Jian S. F. Chen D. L. Huang X. J. Miao J. H. (2021). Organic nitrogen sources promote andrographolide biosynthesis by reducing nitrogen metabolism and increasing carbon accumulation in Andrographis paniculata. Plant Physiol. Biochem. 164, 82–91. doi: 10.1016/j.plaphy.2021.04.016 33975147

[B56] Zhong Y. Li Y. Cheng J. J. (2016). Effects of selenite on chlorophyll fluorescence, starch content and fatty acid in the duckweed Landoltia punctata. J. Plant Res. 129, 997–1004. doi: 10.1007/s10265-016-0848-6 27400684

[B57] Zhu X. C. Song F. B. Xu H. W. (2010). Arbuscular mycorrhizae improves low temperature stress in maize via alterations in host water status and photosynthesis. Plant Soil 331, 129–137. doi: 10.1007/s11104-009-0239-z

[B58] Zou Y. Han C. Wang F. Tan Y. Yang S. Huang C.. (2020). Integrated metabolome and transcriptome analysis reveal complex molecular mechanisms underlying selenium response of *aloe vera* L. J. Plant Biol. 2, 1–9. doi: 10.1007/s12374-020-09285-z

