# Supplementary material for: Response mechanism of major secondary metabolites of Polygonatum kingianum to selenium nanoparticles
Source: Front Plant Sci. 2024 Dec 18;15:1480079. doi: 10.3389/fpls.2024.1480079 (PMC11688289; doi:10.3389/fpls.2024.1480079)
Supplement: Supplementary file 1 [file DataSheet1.zip › Supporting images.docx]

Supplementary Material

## Supplementary Figures


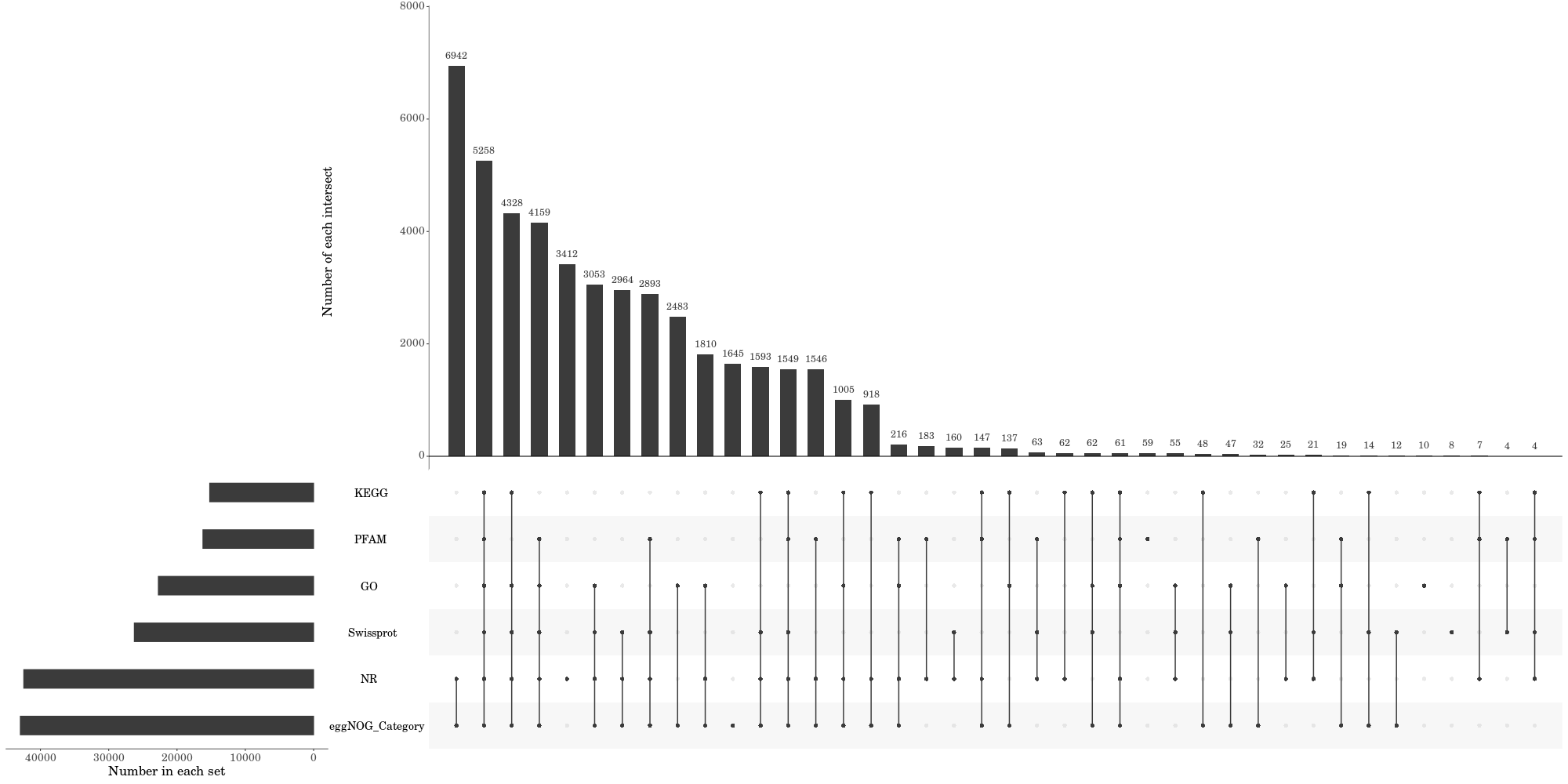


**Supplementary Figure S1.** Annotated information for each database. Number in each set indicates the number of all Unigenes annotated to each database; number of each intersection indicates the number of common Unigenes annotated to multiple databases; a point in the abscissa indicates the number of unique Unigenes annotated to the database; a line connecting multiple points in the abscissa indicates the number of common Unigenes annotated to the multiple databases connected to the line.


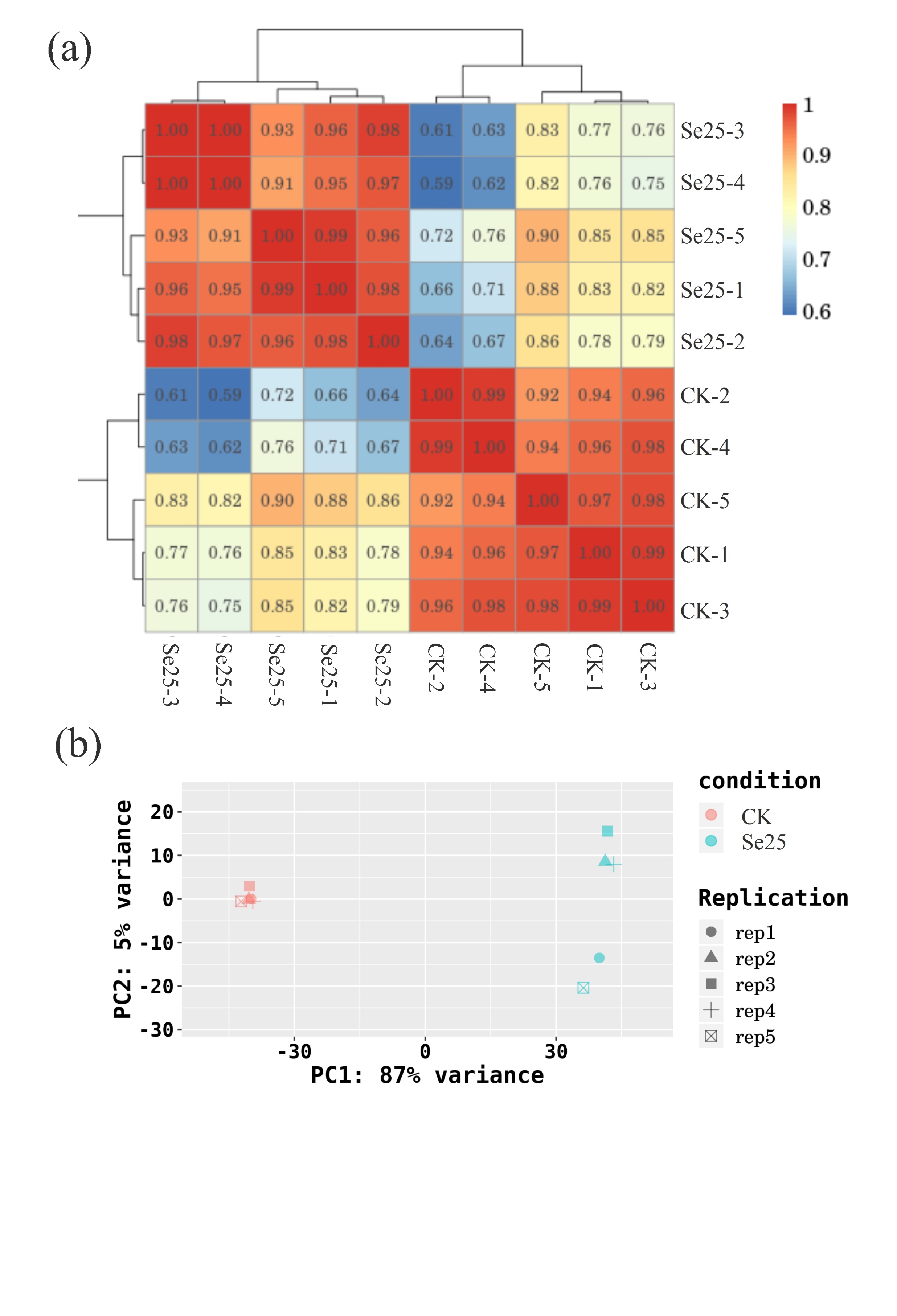


**Supplementary Figure S2** Correlation testing and PCA analysis of transcriptome samples (a) Sample correlation test. The left side and the top side show the sample clustering situation, the right side and the bottom side of the figure show the sample names, and the different colored squares represent the high and low correlation situation of the two samples. (b) PCA Analysis. The horizontal coordinate is the first principal component and the vertical coordinate is the second principal component. Different shapes in the figure indicate different samples and different colors indicate different groupings.


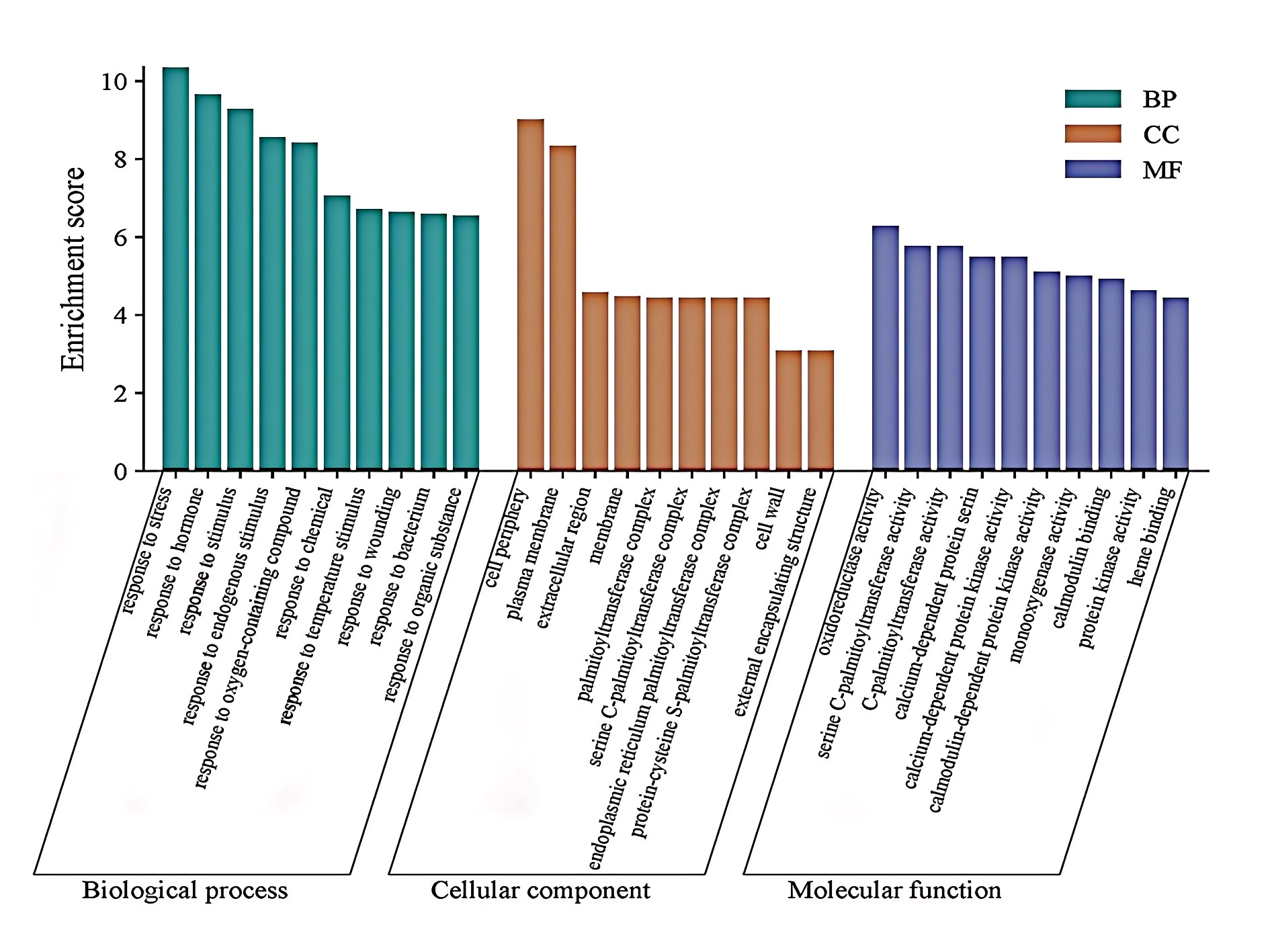


**Supplementary Figure S3** GO pathway enrichment analysis of differential genes.


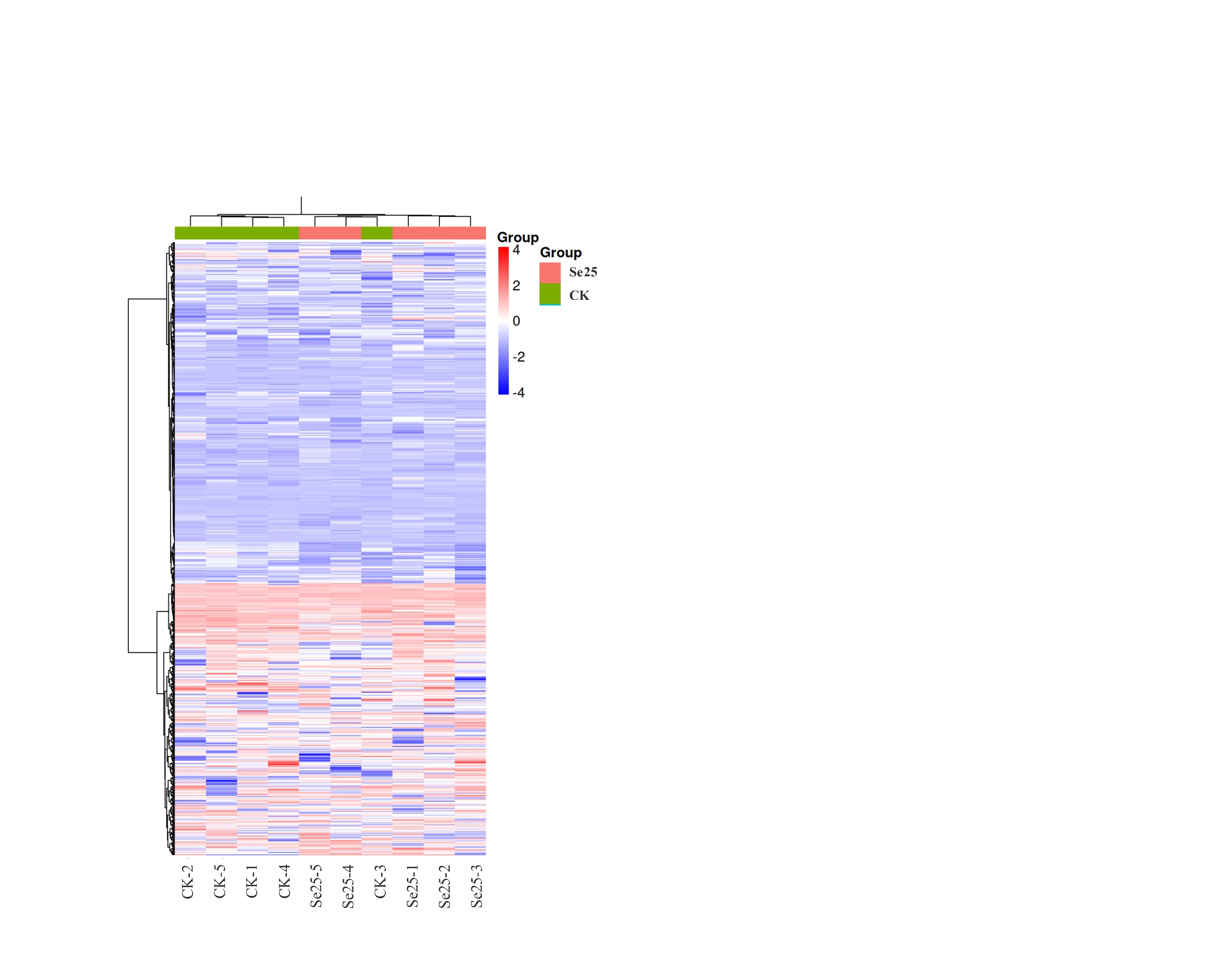


**Supplementary Figure S4** HCA of the overall heat map of metabolites. The left and top sides of the figure show the sample clustering situation, the right side shows the sample name, and the different colored squares represent the high and low correlation situations of the two samples.


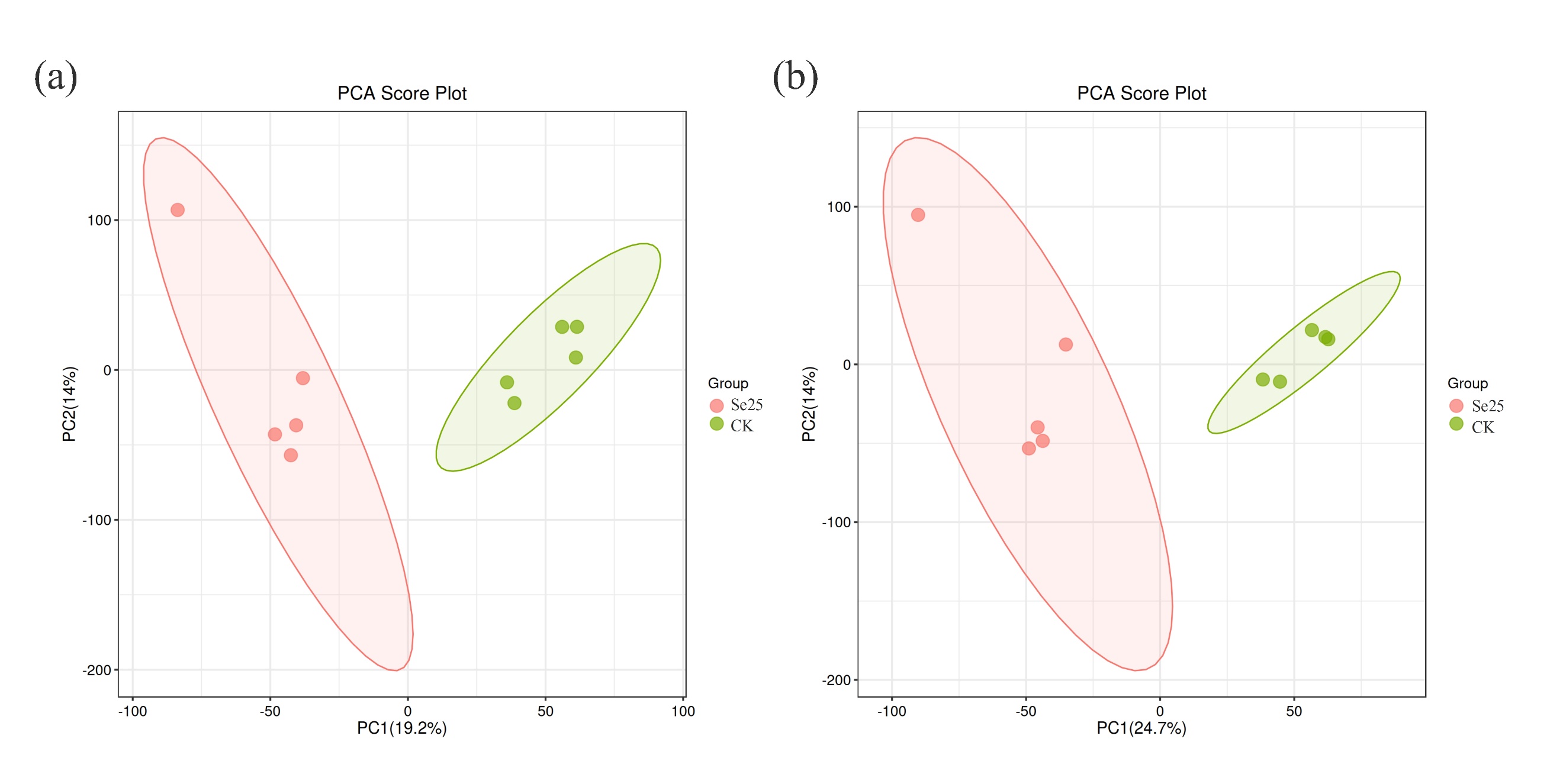


**Supplementary Figure S5** Metabolome PCA analysis. (a) represents the positive ion pattern; (b) represents the negative ion pattern. The horizontal coordinate is the first principal component and the vertical coordinate is the second principal component. Red color represents Se25 treatment and green color represents CK.


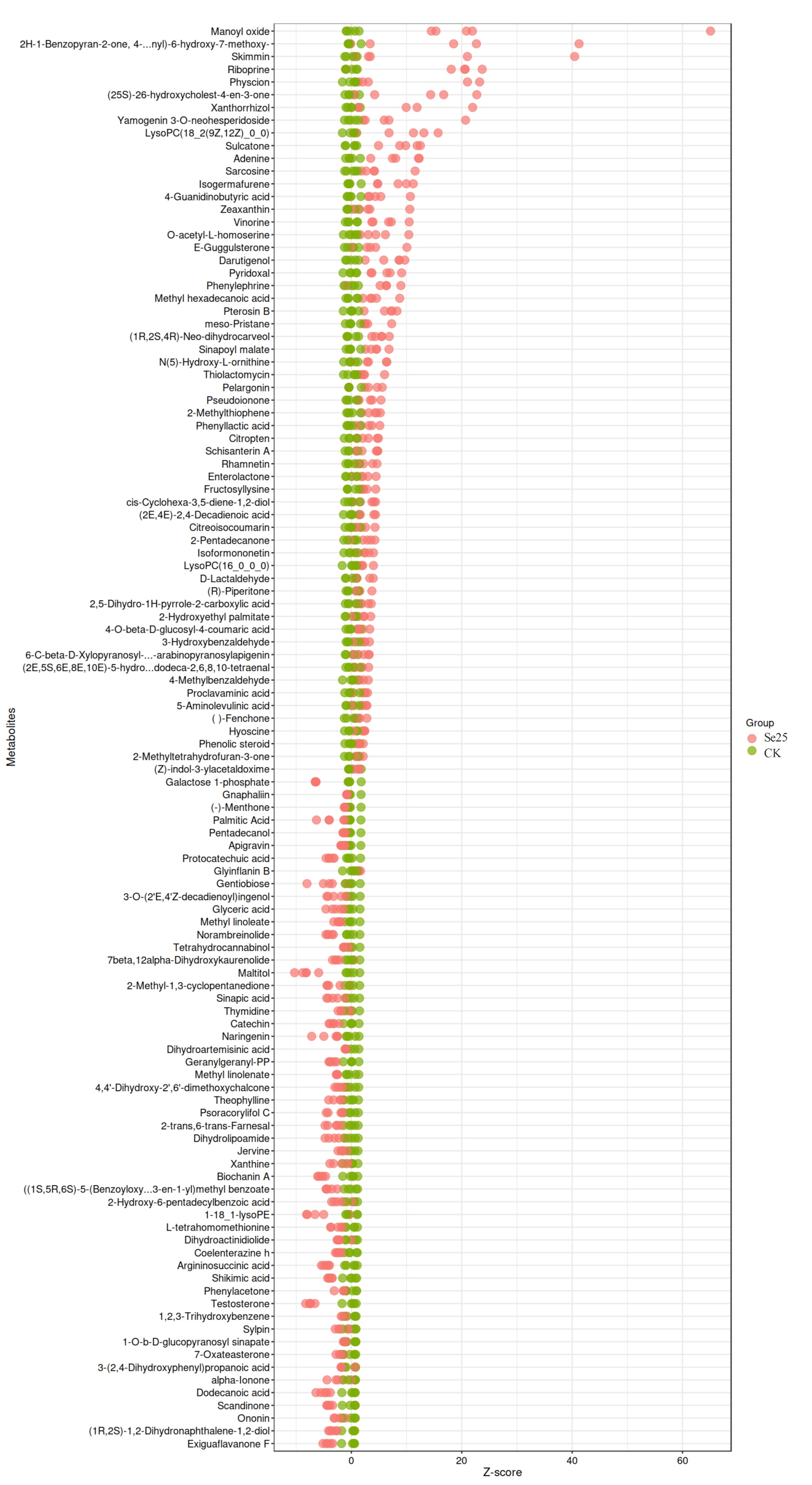


**Supplementary Figure S6** Z-score chart. Horizontal coordinates are converted Z-score values for the relative amount of metabolite in the sample, vertical coordinates are metabolite names. Red represents the Se25 treatment group and green represents CK. the closer to the right, the higher the relative amount of the current metabolite in that sample, and the closer to the left, the lower the amount of the current metabolite.
